# The Antiepileptic Drug Levetiracetam Suppresses Non-Convulsive Seizure Activity and Reduces Ischemic Brain Damage in Rats Subjected to Permanent Middle Cerebral Artery Occlusion

**DOI:** 10.1371/journal.pone.0080852

**Published:** 2013-11-13

**Authors:** Ornella Cuomo, Vincenzo Rispoli, Antonio Leo, Giovanni Bosco Politi, Antonio Vinciguerra, Gianfranco di Renzo, Mauro Cataldi

**Affiliations:** 1 Division of Pharmacology, Department of Neuroscience, Reproductive and Odontostomatologic Sciences, Federico II University of Naples, Naples, Italy; 2 Department of Health Sciences, University Magna Græcia of Catanzaro, Catanzaro, Italy; Rutgers University, United States of America

## Abstract

The antiepileptic drug Levetiracetam (Lev) has neuroprotective properties in experimental stroke, cerebral hemorrhage and neurotrauma. In these conditions, non-convulsive seizures (NCSs) propagate from the core of the focal lesion into perilesional tissue, enlarging the damaged area and promoting epileptogenesis. Here, we explore whether Lev neuroprotective effect is accompanied by changes in NCS generation or propagation. In particular, we performed continuous EEG recordings before and after the permanent occlusion of the middle cerebral artery (pMCAO) in rats that received Lev (100 mg/kg) or its vehicle immediately before surgery. Both in Lev-treated and in control rats, EEG activity was suppressed after pMCAO. In control but not in Lev-treated rats, EEG activity reappeared approximately 30-45 min after pMCAO. It initially consisted in single spikes and, then, evolved into spike-and-wave and polyspike-and-wave discharges. In Lev-treated rats, only rare spike events were observed and the EEG power was significantly smaller than in controls. Approximately 24 hours after pMCAO, EEG activity increased in Lev-treated rats because of the appearance of polyspike events whose power was, however, significantly smaller than in controls. In rats sacrificed 24 hours after pMCAO, the ischemic lesion was approximately 50% smaller in Lev-treated than in control rats. A similar neuroprotection was observed in rats sacrificed 72 hours after pMCAO. In conclusion, in rats subjected to pMCAO, a single Lev injection suppresses NCS occurrence for at least 24 hours. This electrophysiological effect could explain the long lasting reduction of ischemic brain damage caused by this drug.

## Introduction

Levetiracetam (Lev) is a second generation antiepileptic drug structurally related to the nootropic and neuroprotective pyrrolidone compound, piracetam [1]. Beside its potent antiepileptic activity, Lev also has antiepileptogenic effects in electrical [2,3] and audiogenic kindling [4] and in several animal models of epilepsy such as WAG/Rij rats [5,6]. In addition, this drug protects neurons from different types of insults including the intracerebroventricular injection of kainate [7] and brain ischemia induced by middle cerebral artery occlusion (MCAO) [8] or neonatal hypoxia [9]. Lev also reduced brain damage in experimental subarachnoid hemorrhage and closed head trauma [10]. 

The mechanism responsible for Lev-induced neuroprotection and antiepileptogenic effect is unknown. This drug differs from all known antiepileptics for it targets SV2, a protein of the synaptic vesicle fusion complex [11]. Through the interaction with this protein, Lev acts as a general inhibitor of neurotransmitter release [12]. Moreover, we reported that Lev blocks Ca^2+^ release from intracellular IP_3_ stores [13] and a similar effect was observed by others for ryanodine stores [14,15]. Lev also antagonizes the inhibitory effect of Zn^2+^ and β-carbolines on GABA_A_ receptors [16] and has slight inhibitory effects on N-type Ca^2+^ channels [17]. Finally, its major metabolite in humans, 2-pyrrolidinone-n-butyric acid, inhibits hystone deacetylases [18]. All these pharmacological effects could, theoretically, contribute to neuroprotection by a direct effect on neurons [19]. Despite its efficacy in living animals, surprisingly, Lev was ineffective in models *in vitro* of neurodegeneration. Specifically, this drug was unable to protect hippocampal slices from the ischemic damage induced by the combined deprivation of oxygen and glucose [20]. This finding suggests that intact neuronal networks present in the living brain and disrupted by the slicing procedure are necessary for Lev-induced neuroprotection. This hypothesis is in keeping with the evidence that after a focal brain insult, depolarizing waves of spreading depolarization, called post-ischemic depolarizations (PIDs), enlarge the primary lesion by propagating into the surrounding intact brain through preexisting synaptic networks (see 21 for review). At the EEG, PIDs have the electrophysiological features of seizure activity. Because they are not accompanied either by motor or behavioral symptoms, these events are usually defined non-convulsive seizures (NCSs) [22]. Recently, the propagation of NCSs has been directly visualized in the ischemic human brain and the demonstration of their relevance in the progression of ischemic lesions has also been obtained [23]. NCSs also contribute to epileptogenesis accounting for the frequent development of post-ischemic epilepsy in patients surviving stroke [24]. Therefore, a neuroprotective activity in stroke and a protection from the development of postischemic epilepsy could be obtained by suppressing PIDs. In the present paper, we explored whether this electrophysiological effect could be obtained with Lev. To this aim, we examined Lev effect on the generation or propagation of NCSs occurring in rats after the permanent occlusion of the middle cerebral artery (pMCAO), a well known experimental model of brain ischemia.

## Materials and Methods

### Animals

All the experiments were performed in 2-month old, male, Sprague Dawley rats weighing 200-250 g (Charles River, Italy). Rats were group caged on a 12 h light/dark cycle and had free access to food and water. The experimental protocol was approved by the Animal Care Committee of “Federico II”, University of Naples, Italy. Animal housing and experimental procedures were performed according to the recommendations of the guidelines for care and use of experimental animals of The European Community Council directive (86/609/EEC). All efforts were made to minimize animal suffering and to reduce the number of animals used in the experiments. 

### Electrode Implantation

Two/three days before experimental brain ischemia or sham operation, rats were deeply anesthetized by delivering a gas mixture containing 2% sevoflurane in a 70% nitrous oxide/30% oxygen through a nose cone. Then, two burr holes were drilled in each side of the skull, 2 mm laterally to the midline, and 2 and 4 mm posteriorly to bregma to position the EEG recording electrodes onto the dural surface. According to the Paxinos and Watson coordinates, these positions correspond to the parietal region of the neocortex. In addition, a reference electrode was placed posterior to lambda. The electrodes were secured to the skull with acrylic dental cement. Their free ends were soldered to a multipin connector (Plastic One, Roanoke, VA, USA) and the entire assembly was fixed to the skull with dental cement. 

### Basal EEG recordings

For EEG recordings, we used a bipolar recording montage with a ground electrode placed onto the nasal bone. On the day of the experiment, animals were placed in a recording cage equipped with a multichannel swivel commutator (Plastics One, Roanoke, VA, USA). The multipin connector on the rat skull was connected to the swivel system via a flexible shielded cable that allowed free movement of the animal during the EEG recording [25]. The swivel commutator was interfaced through shielded cables with an EEG recording and analysis system (Belight, Galileo NT, EB-Neuro, Florence, Italy). Signals were low-pass filtered at 0.1 Hz before being band-passed between 0.16 and 50 Hz. Data were digitized at 100 Hz, acquired through the analysis software and stored on the hard disk of the computer for offline analysis. 

Basal EEG activity was recorded for 30 minutes, then, the swivel cable was disconnected from the multipin connector on the head of the animal. Lev or vehicle was injected as described in the following section and the rat was deeply anesthetized to be subjected to pMCAO as described below. After the surgical procedure, the animal was returned to its cage and was reconnected to the swivel cable. Twenty minutes after the induction of ischemia, EEG recording was started again and continued for 48 hours. 

### Levetiracetam administration

Lev was dissolved in saline solution and administered at the doses of 50 or 100mg/Kg by a single intraperitoneal (ip) injection. Drug injection and the further experimental procedures were carried out in blind manner. Indeed, the researchers who performed the experiments and analyzed data were not aware of the drug treatment administered to the animal. 

### Experimental brain ischemia

pMCAO was performed using the method described by Cuomo et al. with slight modifications [26]. Briefly, rats were deeply anesthetized with a gas mixture (4% sevoflurane in a 70% nitrous oxide/30% oxygen) delivered through a nose cone. Anaesthesia was then maintained with 2% sevoflurane during the entire surgical procedure. The skin of the neck and the underneath fasciae were sectioned in the sternocleidomastoid region and the neurovascular bundle of the neck was exposed. In sham-operated rats, the surgical section was, then, sutured without proceeding further with arterial occlusion whereas in rats undergoing pMCAO the right carotid bifurcation was exposed and the external carotid artery coagulated distal to the bifurcation and cut. Afterwards, a 6-0 nylon filament was inserted through the external carotid artery stump and advanced into the left internal carotid artery until it blocked the origin of the middle cerebral artery. During this surgical procedure, cortical cerebral blood flow (CBF) was continuously monitored with a laser-doppler flowmeter (Periflux system 5000, Perimed, Milan Italy) [27]. Once a stable drop in blood flow signal was obtained ipsilaterally to the occlusion, the filament was secured to the vessel by ligation. The filament was left in place to permanently occlude the middle cerebral artery. Body temperature was monitored throughout the entire duration of the surgical procedure and maintained at 37.5 °C with a thermostatic blanket. Arterial blood gases were measured before and after ischemia through a catheter inserted into the femoral artery (Rapid lab 860; Chiron Diagnostic, Emeryville, California, USA). Only the animals showing a reduction in CBF of at least 70% were considered ischemic and entered the further steps of the experimental protocol. No significant difference in CBF was found at the retrospective analysis of the laser-doppler flowmeter data between among the different experimental groups (data not shown). 

### Evaluation of neurological deficits and of the ischemic volume

Neurological deficits were evaluated 24 or 72 hours after pMCAO according to Clark’s scale [28]. The statistical significance of the difference in the scores obtained in the different groups of animals was assessed by non-parametric analysis using the Nemenyi test. The threshold for statistical significance was set at p<0.05.

The extension of the ischemic lesion was assessed 24 or 72 hours after pMCAO as described elsewhere [29]. Briefly, rats were sacrificed by decapitation. Brains were quickly removed, placed in ice-cold saline solution for 5 minutes and then cut into 500 µM coronal slices with a vibratome (Campden Instrument, 752M; UK). Sections were incubated for 20 minutes in a saline solution containing 2% Triphenyl tetrazolium chloride (TTC) and, then, fixed in 10% formalin overnight. The infarcted region in each slice was identified as the white area after TTC staining. The surface areas of the entire slice and of the ischemic lesion were measured using the image analysis software Image-Pro Plus 4.1 (Media Cybernetic, Rockville, MD, USA) in all the slices between +4.7 and -4.9mm from bregma according to the Paxinos atlas. These coordinates define the brain region that usually includes the entire ischemic lesion caused by pMCAO. The values obtained were used to calculate the volumes of the hemisphere and of the infarcted area in this specific region. The volume of the ischemic area was, then, expressed, as percentage of the entire volume of the infarcted hemisphere. By this approach, the data were corrected for brain edema. The statistical significance of the difference between the experimental groups was evaluated by one-way ANOVA followed by the Newman-Keuls post hoc test. A p value <0.05 was considered statistically significant.

### EEG analysis

Qualitative EEG analysis was performed off-line by visual examination. By using this approach, we distinguished periods of normal activity, of reduced electrical activity, in which the traces became close to the isoelectric, and NCS events. All the rhythmic discharges with spike components that persisted >10 sec were classified as NCS [22,25]. NCSs were further subdivided according to their shape into single spikes, spike-wave, poly-spike or polyspike-waves discharges. The number, frequency and amplitude of all these events were computed in each of the experimental groups.

Quantitative EEG analysis was performed using the Fast Fourier Transform (FFT) and a commercial software (Belight, Galileo NT;EB-Neuro, Florence, Italy software). Specifically, the bipolar signal from each neocortical area in both brain hemispheres was processed using Fast Fourier Transform and its main spectral components were separated. Specifically, four main band components were identified in the frequency range between 0.25 and 32 Hz: delta (0.25–3.9 Hz), theta (4.0–7.9 Hz), alpha (8.0–12.9 Hz) and beta (13.0–30 Hz). Both in control and in Lev-treated rats, the mean power (in μV^2^) of each of these components was computed by integration of the power spectra in the respective frequency ranges. 

All the EEG data are presented as mean±SEM. Statistical analysis of the results of qualitative and quantitative EEG analysis was performed by one way ANOVA, followed by the post-hoc Tukey-Kramer test for multiple comparisons. The threshold for significance was set at p<0.05. Computational analysis and signal processing were performed using the GraphPad Prism 5.0 software (GraphPad Inc., La Jolla, CA, USA). 

### Drugs and chemicals

Lev was kindly provided by Dr. B. Ferrò (UCB, Italia). Sevoflurane was from Abbott (Abbott Park, Illinois, USA) whereas oxygen and nitrous oxide were from Air Liquide Italia (Rome, Italy). All chemicals were of analytical grade and were purchased from Sigma Aldrich Italia (Gallarate, Milan, Italy). 

## Results

### Effect of Levetiracetam on ischemic brain damage in rats subjected to pMCAO

**Figure 1:**
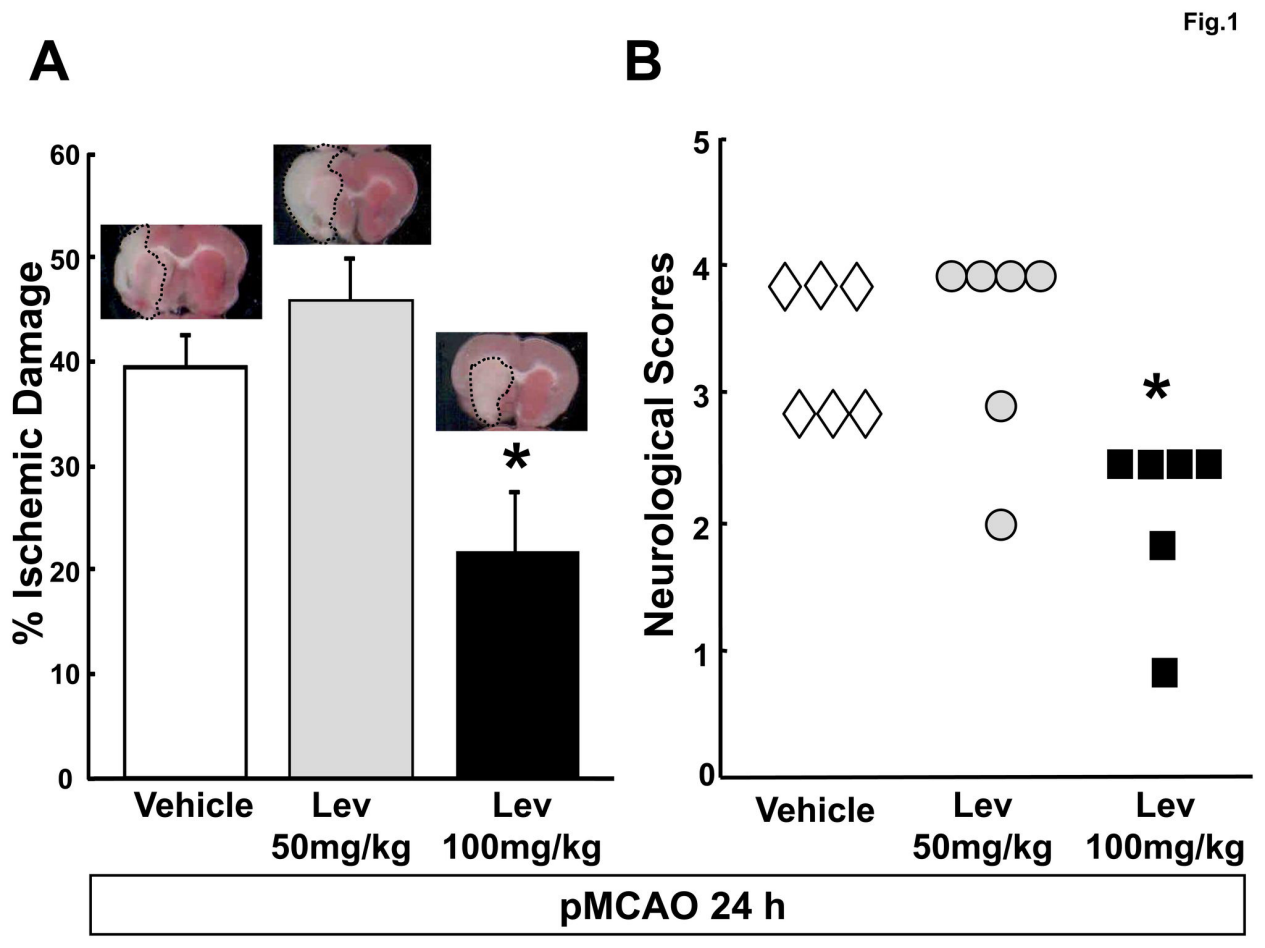
Levetiracetam reduces the ischemic brain damage evaluated 24 hours after pMCAO. A, percent ischemic damage in vehicle- and Lev-treated ischemic rats. The bar graph reports the mean+SEM of the volume of the ischemic lesion normalized to the volume of the entire hemisphere ipsilateral to vessel occlusion. See the methods section for more details. The microphotographs on the top of the bars show representative TTC-stained brain slices obtained 24 after pMCAO from rats belonging to the respective experimental groups. Note that the ischemic area is smaller in the animal receiving the highest dose of Lev than in the other two rats. B, neurological deficits in vehicle and Lev-treated ischemic rats, evaluated 24 hours after pMCAO. The scatter graph shows the individual data of the Clark’s score for generalized neurological deficits in rats receiving vehicle, 50mg/kg or 100 mg/kg Lev as indicated. *, p<0.05 vs vehicle (n=6 in all groups).

We evaluated the effect of two different Lev concentrations, 50mg/kg and 100mg/kg on the development of ischemic brain damage and on the appearance of neurological symptoms after pMCAO. A first group of Lev-treated and control animals were sacrificed 24 hours after pMCAO to assess the severity of the ischemic lesion caused by vessel occlusion. In control rats, we observed a large brain infarction that involved the majority of the temporo-parietal cortex in the brain hemisphere ipsilateral to pMCAO. This lesion extended from the cortex through the hemispheric white matter till the striatum. In the region between +4.7 and -4.9mm from bregma, about 40% of the brain hemisphere was infarcted (Figure 1A). The normalized volume of the ischemic brain was similar to that of controls in rats receiving 50mg/Kg Lev and significantly smaller in those injected with 100mg/kg Lev (39.5 ± 3.1, 45.7 ± 4.2 and 21.3 ± 6.3 % of the whole hemisphere in controls and in rats receiving 50 or 100 mg/kg Lev, respectively, n=6 for each group, p<0.05 100mg/Kg vs 50mg/kg Lev and vs controls) (Figure 1A). Relevant neurological symptoms appeared in control animals after brain ischemia, including abnormal posture and reduced spontaneous locomotor activity. These clinical manifestations were markedly attenuated in rats treated with 100 mg/kg Lev but not in those receiving the lower dose of 50mg/kg. Accordingly, Clark’s score was significantly lower in rats receiving 100mg/kg Lev respect to the other two groups of animals (median of deficit scores: 3.5 for vehicle treated rats vs 3 for 100mg/kg Lev treated rats, p<0.05, n=6) (Figure 1B). We never observed motor or behavioral seizures neither in controls nor in Lev-treated animals.

**Figure 2:**
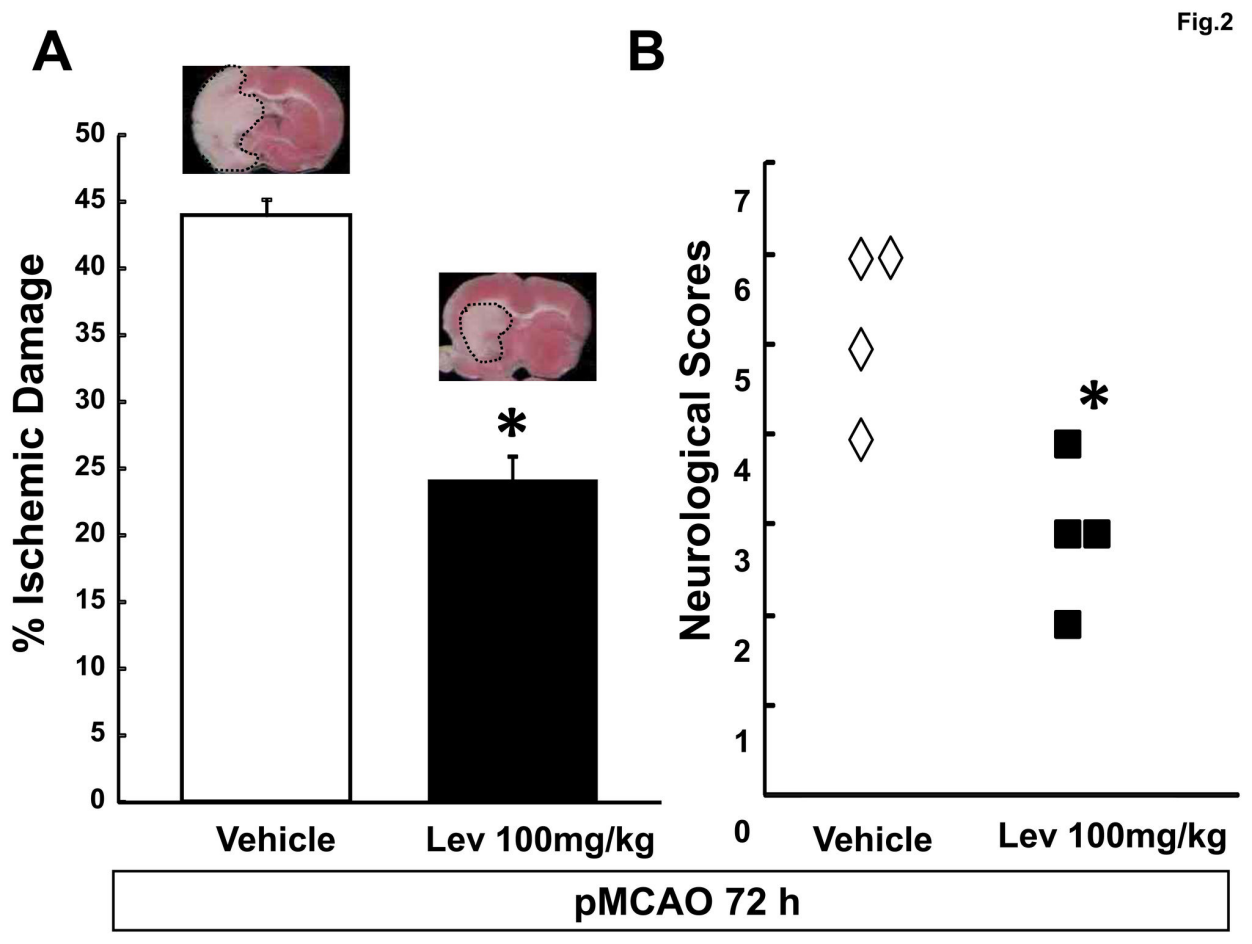
Levetiracetam neuroprotection persists 72 hours after pMCAO. A, percent ischemic damage in vehicle and Lev-treated ischemic rats. The bar graph reports the mean+SEM of the volume of the ischemic lesion normalized to the volume of the entire hemisphere ipsilateral to vessel occlusion. See the methods section for more details. The microphotographs on the top of the bars show representative TTC-stained brain slices obtained 72 after pMCAO from rats belonging to the respective experimental groups. Note that the ischemic area is smaller in the animal receiving Lev than in the control rat. B, neurological deficits evaluated 72 hrs after pMCAO in vehicle and Lev-treated ischemic rats. The scatter graph shows the individual data of the Clark’s score for generalized neurological deficits in rats receiving vehicle, or 100 mg/kg Lev as indicated. *, p<0.05 vs vehicle (n=6 in all groups).

 A second group of controls and of rats receiving 100mg/kg Lev were sacrificed 72 hours after pMCAO to establish whether the neuroprotection elicited by a single injection of Lev still persisted at that time. Normalized volume of the ischemic lesion (43.9 ± 1.1 vs 24.1 ± 1.9 % of the whole hemisphere in controls and 100mg/kg Lev treated rats, p<0.05, n=6 in both groups) and Clark’s neurological scores (median of deficit scores: 5.5 vs 3 in control and 100mg/kg Lev treated rats, respectively) were still smaller in Lev treated rats than in controls 72 hours after pMCAO (Figure 2A and B). 

### Effect of Levetiracetam on the non convulsive seizure discharges occurring after pMCAO

**Figure 3:**
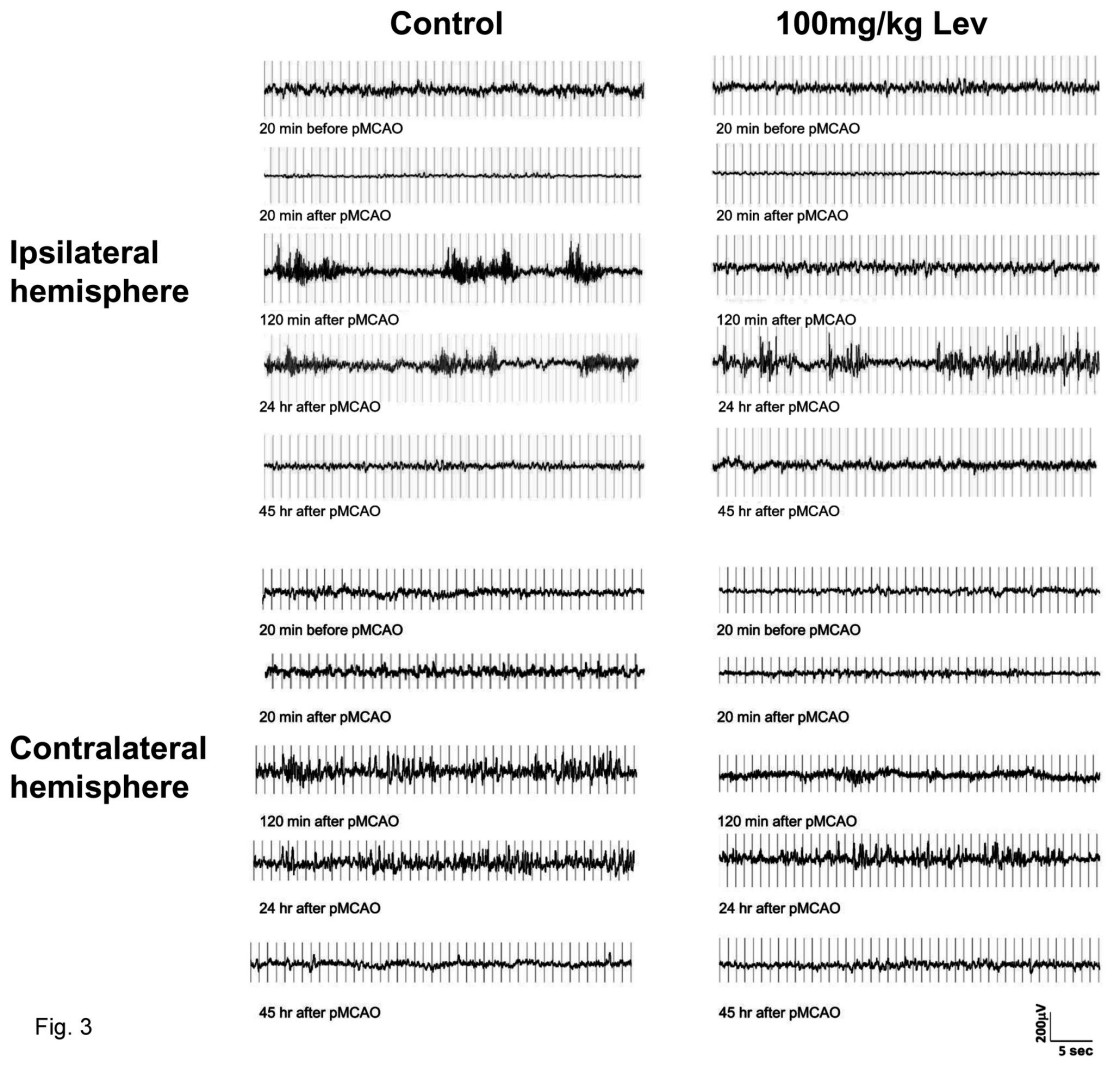
Effect of brain ischemia and of Levetiracetam on EEG activity. The figure reports EEG recording samples of about 40 sec in duration obtained in a representative control (on the left) and a Lev-treated rat (on the right) before and after pMCAO. For each animal, traces obtained immediately before, and 20 min, 120 min, 24 and 45 hours after pMCAO are reported. The traces shown in the panels on the top of the figure were recorded ipsilaterally to MCAO whereas those in the bottom panels are from the contralateral side. Note that EEG activity was markedly suppressed after pMCAO in the ipsi- but not in the contralateral brain hemisphere. Note also that a significant EEG activity reappeared, in the form of NCSs, 120 min after pMCAO in the control rat and only 24 hours after vessel occlusion in the Lev-treated rat. In the traces from the contralateral hemisphere reported in the bottom panels, IRDAs can be easily identified as brief bursts occurring in isolation. Note that these events are already large and well defined in traces obtained 120 min after pMCAO in control but not in Lev-treated rats in which they become clearly evident only after 24 hours.

**Figure 4:**
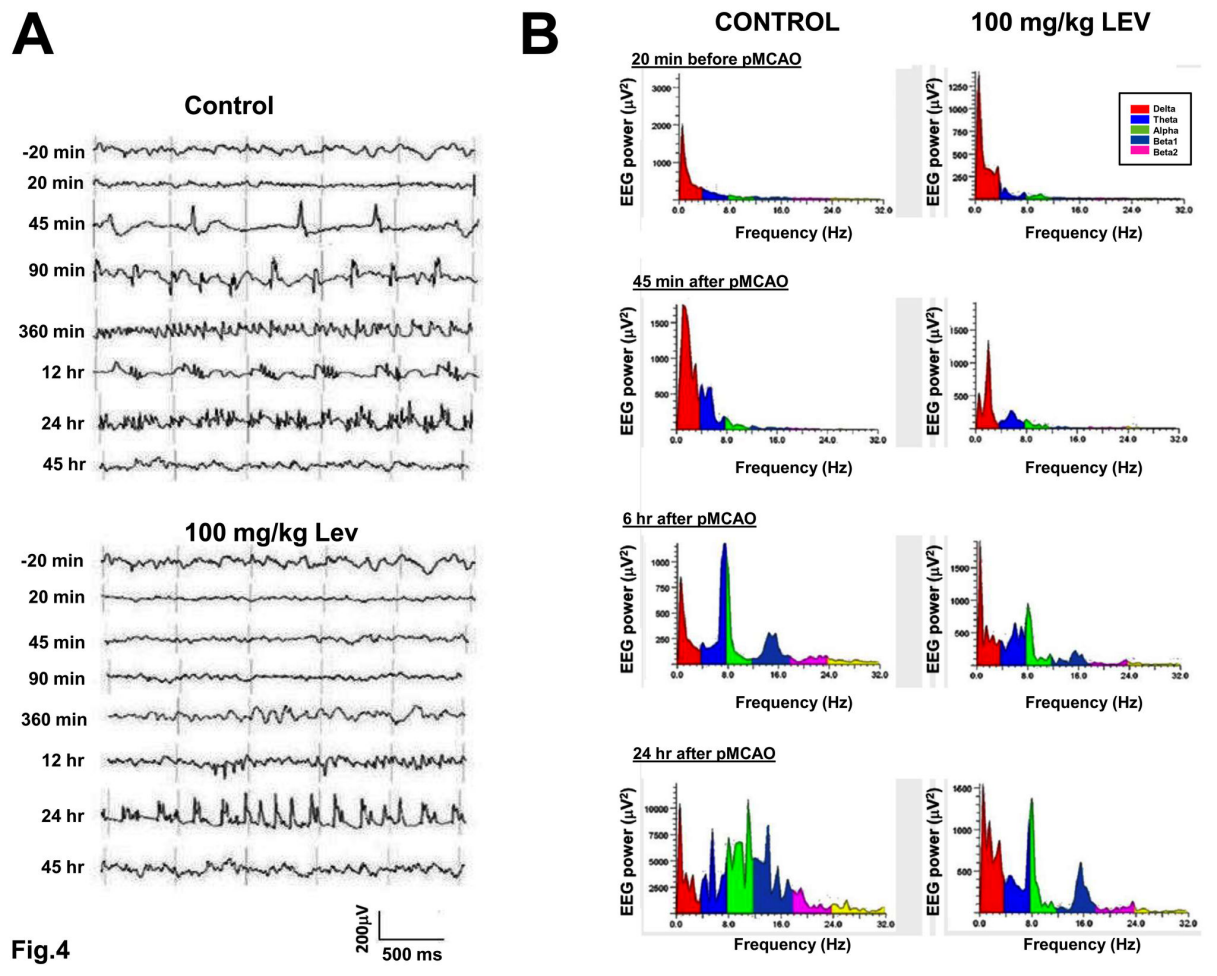
Time course and electrophysiological characteristics of the non-convulsive seizures in control rats and in rats treated with Levetiracetam. A, examples of the different types of EEG activity recorded at different times after pMCAO in a control (top) and in a Lev-treated (bottom) rat. Note the different time course of NCSs in the control and in the Lev-treated rat. In the control rat, spike-wave activity predominated 45 and 90 min after pMCAO, polyspike-waves after 12 hours and polyspikes after 24 hours. In the Lev-treated rat, NCSs appeared only 12 hour after pMCAO in the form of polyspike complexes. B, Spectral graph analysis of the EEG traces obtained at different times from pMCAO in a control rat (top) and in a rat injected with 100mg/kg Lev. The graphs show the frequency distribution of the power of the EEG signal in the frequency range from 0.25 up to 32 Hz. The main band components of the EEG signal, delta, theta, alpha, beta 1, beta 2, are shown in different colors as detailed in the insets. Each spectral graph was obtained by the analysis of 10 sec of artifact-free recordings. Note that, both in the control and in the Lev-treated rat, the appearance of NCSs corresponded to a strong increase of the power in the regions of high theta and low alpha bands.

To establish whether the neuroprotective dose of Lev (100mg/kg) affected NCS generation or propagation we performed EEG recordings in ischemic rats receiving this dose of the drug or its vehicle. In addition, EEG activity was also monitored in two different groups of sham-operated rats, the first ip-injected with Lev and the second with its vehicle. Baseline EEG activity was recorded in all the animals for 30 min before Lev or vehicle injection. In these awake and freely moving animals, visual inspection of the EGG traces showed a predominance of slow activity (Figure 3 and Figure 4A). Power spectra analysis confirmed that the majority of brain electrical activity was in the delta frequency range (Figure 4B). 

**Figure 5:**
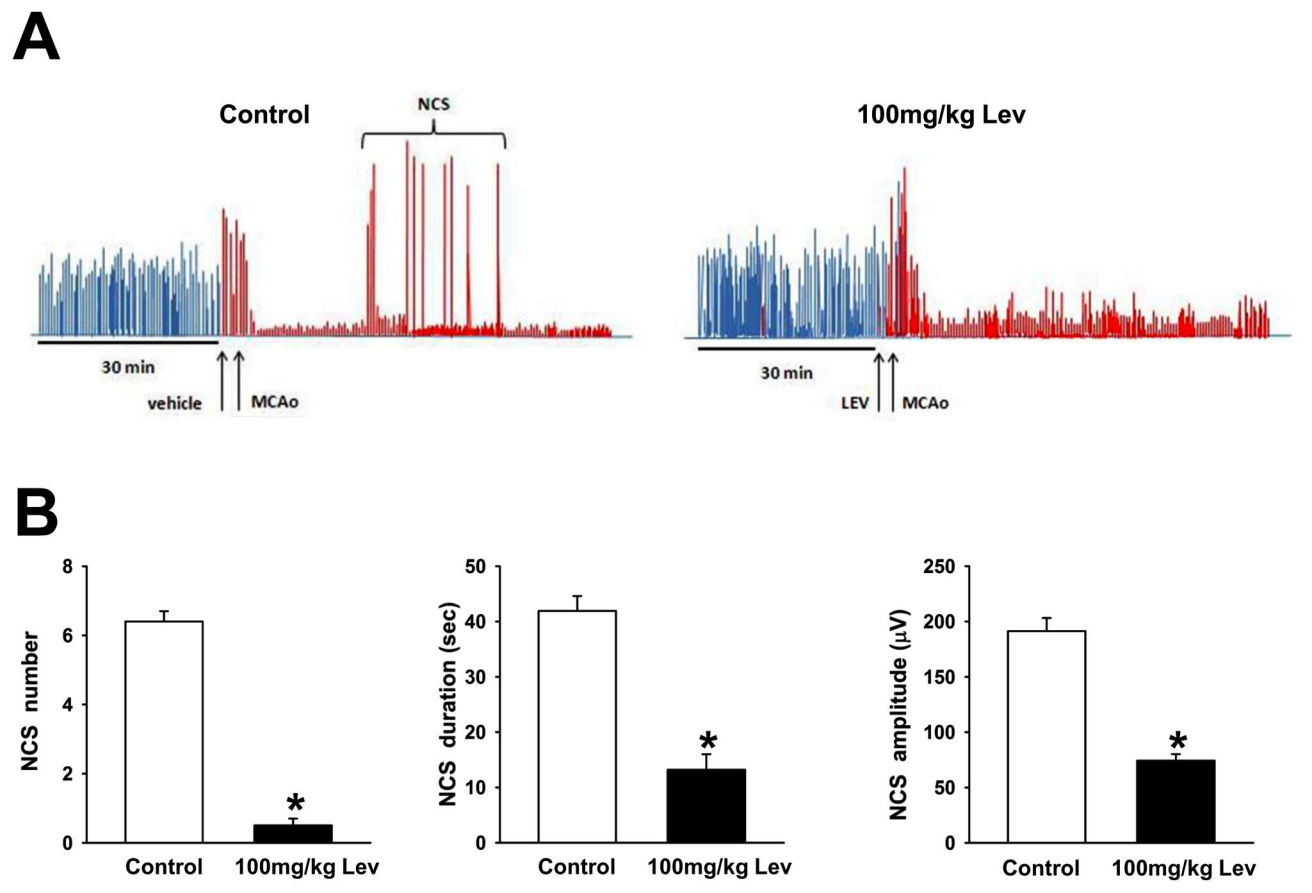
Levetiracetam prevents the occurrence of non convulsive seizures. A, representative spectral trend graphs showing the changes in total EEG power after pMCAO in a control (left) and in a Lev-treated (right) rat. Each bar represents the total power (in μV^2^) calculated by integration of the power spectrum of the EEG recorded during about 120 min interval. The bars colored in red denote the data obtained during the first 90 min of recordings after pMCAO. Note the dramatic drop in total EEG power after pMCAO induction both in the presence and in the absence of Lev. The sudden and transient increases in EEG power correspond to the NCS activity that appears in the control but not in the Lev-treated rat. B, Number, duration and amplitude of NCSs in Lev treated and in control animals. The bar graphs show from the left to the right the mean+SEM of the number, duration in sec and amplitude in μV of the NCS observed during the first 2 hours after pMCAO in control (n=7) and Lev-treated (100mg/kg; n=7) rats. * p<0.05.

Both in rats receiving Lev and in controls, baseline EEG pattern was dramatically modified after brain ischemia. Conversely, no change in EEG activity was observed in sham-operated rats (data not shown). In rats subjected to pMCAO, when EEG recording was started again, 20 min after vessel occlusion, spontaneous cortical electrical activity was greatly reduced respect to baseline in the ischemic but not in the contralateral brain hemisphere where it remained fairly stable (Figure 3 and Figure 4A). Consequently, in both groups, the total power of the spectrum of the EEG recorded ipsilaterally to MCAO decreased down to about 40% of basal values and a general decrease in EEG power occurred in the spectral trend graph (Figure 5). No significant difference was observed between Lev-treated and control rats in the extent of this ischemia-induced suppression of the EEG activity. No change was observed in the percent representation of the different EEG spectral components neither in control nor in Lev-treated rats. In the early phases after pMCAO, thus, the EEG spectrum still showed a large prevalence of delta activity. On the contrary, significant differences emerged, thereafter, between rats receiving 100 mg/kg Lev and controls. Indeed, in controls but not in rats treated with 100 mg/kg Lev, a paroxysmal electrical activity appeared in the ischemic brain hemisphere by 30-45 min after pMCAO. This resurgent electrical activity differed from that observed before the induction of ischemia and was dominated by epileptiform discharges whose pattern progressively changed over the following 6-12 hours. The first events that we observed were rhythmic spikes (Figure 4A). Mean amplitude, duration and frequency of these events were 191.3 ± 11.9 μV, 41.9±2.7 sec, and 2.5 ± 0.9 Hz, respectively. Single spikes were gradually replaced by spike-wave and polyspike complexes that represented the prevalent electrical discharges 120 min after pMCAO (Figure 4A). Twenty four hours after the induction of pMCAO, EEG activity was virtually composed only by arrhythmic polyspikes (Figure 4A). At spectral analysis, NCSs corresponded to the appearance of a distinct power peak in the regions of high theta and low alpha activity with its maxima around 8 Hz (Figure 4B). In the spectral trend graph NCSs appeared as brief high power episodes superimposed on baseline (Figure 5A). These discharges lasted approximately 45 hours. Afterwards, the EEG pattern returned to normal.

Lev (100mg/kg) had a dramatic effect on the EEG response to pMCAO but it did not modify background EEG activity in sham-operated rats (data not shown). Specifically, this drug reduced NCS occurrence (Figure 3 and Figure 4A). While in control rats a mean of 6.4 + 0.3 NCSs occurred during the first two hours of recordings after pMCAO, only 0.5 + 0.2 events were observed in Lev-treated animals (p<0.001). In this group of rats, NCSs were also shorter (13.2+2.8, vs 41.9+2.7 sec, p<0.01, n=7) and their mean amplitude was lower than in controls (74,3+5,9 vs 191,3+11,9 μV, p<0.01) (Figure 5B). Approximately 6 hours after Lev injection and pMCAO induction, EEG activity begun to increase in Lev-treated animals (Figure 4A). The first EEG events observed in Lev-treated rats were sharp-waves. They were followed by single spikes and, then, by spike and wave, polyspikes and polyspikes and wave (Figure 4A). At power spectra analysis, the main spectral component peaked at approximately 8 Hz, encompassing both theta and alpha activity (Figure 4B). Therefore, this epileptiform activity recapitulated the electrical events occurring at earlier stages in control animals. These events had, however, a power significantly smaller than those observed in controls at the same time (23738.0+1306.0 vs 7675.0+255.6 μV^2^, p<.0.01, n=7). In Lev-treated rats, as in controls, epileptiform discharges disappeared approximately 45 hours after pMCAO. 

Both in control and in Lev-treated ischemic rats, EEG activity was modified not only ipsilaterally but also contralaterally to pMCAO. Specifically, an intermittent rhythmic delta activity (IRDA) appeared in control rats by 45 min from ischemia induction (Figure 3). This activity consisted of bursts in the delta range lasting less than 10 sec that occurred more often in isolation. IRDAs were still observed 24 hours after MCAO whereas they completely disappeared by 45 hours. IRDAs also occurred in rats treated with 100 mg/kg Lev but, when compared 120 min from pMCAO, to those recorded at the same time in control animals, they had a smaller amplitude (97.3±8.3 vs 116.5±7.4 μV, p<0.01, n=7), lasted less (1.3±0.2 vs 4.53±0.8 sec, p<0.01, n=7) and their frequency was lower (0.3±0.1 vs 1.24±0.1 Hz, p<0.05, n=7). As in control also in Lev-treated rats IRDAs disappeared by 45 hours from MCAO (Figure 3).

## Discussion

The main finding of the present study was that the antiepileptic drug Lev prevented the appearance of NCSs in the parieto-temporal cortex of rats subjected to pMCAO. Parallel to this electrophysiological effect a significant reduction in the ischemic brain damage caused by this surgical procedure also occurred.

Since the seminal work of Leao [30] it has been established that brain ischemia causes relevant changes in cortical electrical activity both in men and in experimental animals (see 21 for review). In accordance, in our control group, we observed prominent changes in EEG activity after experimental ischemia. The alterations that we observed are similar to those described by Lu and coworkers [31] who reported a very detailed topographical and spectral analysis of the electrical events originating in the cerebral cortex of rats subjected to MCAO. In particular, in our experiments, a profound suppression of cortical electrical activity appeared in rats subjected to pMCAO as early as 15-20 min after the initial insult in the ischemic but not in the contralateral brain hemisphere. The evidence that no suppression of background EEG activity did occur in sham-operated rats further supports the idea that it was actually caused by brain ischemia and it was not a mere consequence of surgery and/or anesthesia. Ischemia-induced loss of EEG activity is currently considered the consequence of a non-spreading depression of the synaptic activity caused either by the impairment of vesicular release of excitatory neurotransmitters [32] or by the massive release of adenosine in the lesioned brain [33]. Approximately 45 min after the induction of ischemia, we observed a resurgent but abnormal EEG activity in the form of NCSs on the side of middle cerebral artery occlusion. Interestingly, the electrophysiological characteristics of these events gradually changed over time. Specifically, whereas during the first 90 min the EEG activity essentially consisted of single high voltage and low frequency spikes, spike-wave discharges became prevalent after 360 min. Brain ischemia also induced the appearance of an abnormal EEG activity in the brain hemisphere contralateral to pMCAO. This activity consisted of brief bursts in the delta frequency range and was similar to IRDAs described by Hartings et al. in experimental brain ischemia in the rat [25]. Its genesis is still controversial but it could be an indirect consequence of the damage occurring in far brain regions being caused, for instance, by subtle dysfunction in midline cerebral structures due to the increase in intracranial pressure [25].

A single intraperitoneal injection of Lev at the dose of 100 mg/kg virtually abolished both NCSs and IRDAs though this effect was only transient. In animals receiving the drug at this concentration EEG remained markedly suppressed till 24 hours after pMCAO when an epileptiform activity appeared in Lev-treated rats. This activity became, then, progressively more intense though it was significantly lower than in controls. This gradual reappearance of activity can be easily explained by pharmacokinetics considerations. The half life of the drug in the rat is, indeed, approximately 3 hours in blood and 5 hours in cerebrospinal fluid [34]. 

The mechanism responsible for the suppression of postischemic electrical activity by Lev in both the cortex ipsi and contralateral to pMCAO remains to be determined. However, it could be dependent on the ability of this drug to suppress neurotransmitter release [12] either by interacting with the vesicular protein SV2 [11] that controls the priming of synaptic vesicles for fusion [35-37], or by decreasing Ca^2+^ release from the intracellular stores [38]. The integrity of glutamatergic neurotransmission seems, indeed, to be essential for the propagation of spreading depolarization waves in experimental brain ischemia in the rat [39]. These events that can be measured as NCSs at EEG are generated at the border between the core and the penumbra of the ischemic lesion when the anoxic depolarization invades the still alive brain tissue of the latter region (see 21 for review). This depolarization wave, presumably, activates local brain networks along which NCSs are then propagated. In accordance with this theory, NCSs are significantly increased in transgenic mice overexpressing mutated, hyperfunctional forms of the P/Q type channels, the predominant form of voltage-gated Ca^2+^ channels responsible for neurotransmitter release in the glutamatergic terminals [40]. Conversely, they can be blocked by inducing a general inhibition of neurotransmission with the simultaneous application of blockers of AMPA/kainate and NMDA receptors and of voltage gated Na^+^ and Ca^2+^ channels [41]. The need of a marked suppression of neurotransmitter release for a significant NCS suppression could explain why not all the antiepileptic drugs are effective against these events [22] that are considered especially sensitive only to compounds acting on multiple targets [42]. 

In our experiments, the brain damage induced by ischemia was significantly smaller in the animals that received Lev than in controls. These data resemble those reported by Hanon and Klitgaard [8] in rats subjected to the transient occlusion of the middle cerebral artery. Surprisingly, in brain slices undergoing oxygen and glucose deprivation in vitro, Lev did not reduce the ischemic damage evaluated either as propidium iodide staining [20] or as suppression of field electrical responses to extracellular stimulation [43]. Moreover, this drug did not suppress the spreading depression elicited by oxygen and glucose deprivation in rat cortical brain slices in vitro [44]. The inconsistency between the data obtained *in vitro* and in living animals suggests that Lev needs intact brain networks to exert its favourable effects in ischemia. This fits well with the idea that this drug could suppress the synaptic propagation of NCS. Electrocorticographic and fMRI data show, indeed, that both in humans and in rodents these depolarizing waves cycle around the ischemic core for several days after the ischemic insult [23]. We also have to remind that, recently, Meehan and co-workers [12,45] showed that Lev has to enter the synapse through the recycling vesicular route to exert its antiepileptic activity. This implies that a certain degree of synaptic activity could be needed to see Lev-induced neuroprotection. Therefore, we can speculate that the lack of neuroprotection in the preparations *in vitro* is the result of their low or absent spontaneous network activity. A significant neuroprotection *in vivo* despite the lack of a neuroprotective effect *in vitro* has been reported also for gabapentin and ethosuximide [46]. Intriguingly, also these compounds significantly suppressed NCS occurrence [46].

In conclusion, we showed that Lev suppresses NCSs in an animal model of stroke. Because NCSs contribute to enlarge the ischemic lesion [23] our findings could help explaining the neuroprotective activity of this drug. Considering that NCSs also have a role in post-stroke epileptogenesis [24], the ability of Lev to suppress these electrical events adds new arguments to suggest that this drug could be useful for the prophylaxis of late post-stroke seizures. This form of epilepsy represents a major problem in clinical neurology as it occurs in 11.5% of the patients surviving stroke [47] and accounts for the more prevalent form of acquired epilepsy in adults [48,49]. While other antiepileptic drugs like ethosuximide, gabapentin, topiramate, phosphenytoin, and valproate also suppress NCSs after MCAO [22,50], the better safety profile of Lev respect to these older drugs warrants further studies to validate its early use in stroke patients. 
